# Frederick George Farr

**Published:** 1924-07

**Authors:** 


### FREDERICK GEORGE FARR.

THE Bristol Medical Library and all those who use it have sustained a most serious loss by the death of our highly-valued and esteemed chief clerk, Mr. Frederick George Farr, at the early age of 43 years. He began as assistant there, and then for a time he was in the Arts Library, and in 1903, when Mr. Statton left, he became chief clerk to the Medical Library. His knowledge of the books, pamphlets and periodicals in the library was remarkable, and when references were given by readers he was most careful and painstaking in searching out the matter required. He will be especially missed by all those frequenting the library as readers or for special and research work, for he was always ready to place his extensive knowledge of the varied contents of the library and the possibility of out-of-the-way references on a given subject at the service of all who sought his aid. His experience of the various matters and details involved in library management and arrangement was large, and it was of the greatest help in the recent moving of the books to the new Medical Library in the south wing of the University.

During the war he served for a time in the army.

He leaves a widow and two daughters, and was interred at Henbury after a service at St. Edyth's Church, Sea Mills, where he was a sidesman.

The Hon. Medical Librarian wishes to express his keen appreciation of the great help the deceased has always been to him in the various phases of library work, always carrying out his instructions and wishes with care and skill. For some years past he has acted as his Secretary, and the Hon. Librarian has always looked upon him as a personal friend as well as a most capable colleague.

Apart from his library duties, the Secretaries of the *Medico-Chirurgical Journal* and various Medical Societies gratefully remember the invaluable work he did with regard to the meetings, the *Journal,* and the lists of members.

